# Severe Odontogenic Infections during Pregnancy and Related Adverse Outcomes. Case Report and Systematic Literature Review

**DOI:** 10.3390/tropicalmed6020106

**Published:** 2021-06-21

**Authors:** Resi Pucci, Andrea Cassoni, Daniele Di Carlo, Marco Della Monaca, Umberto Romeo, Valentino Valentini

**Affiliations:** 1 Department of Oral and Maxillofacial Sciences, Sapienza University of Rome, via Caserta 6, 00161 Rome, Italy; resi.pucci@uniroma1.it (R.P.); andrea.cassoni@uniroma1.it (A.C.); marco.dellamonaca@uniroma1.it (M.D.M.); umberto.romeo@uniroma1.it (U.R.); valentino.valentini@uniroma1.it (V.V.); 2 Oncological and Reconstructive Maxillo-Facial Surgery Unit, Policlinico Umberto I, Viale del Policlinico 155, 00161 Rome, Italy

**Keywords:** odontogenic infection, dental abscess, life-threatening odontogenic complications, pregnancy, adverse pregnancy outcomes

## Abstract

Odontogenic infections have the potential to develop into deep-space infections and may cause severe diseases with possible life-threatening complications. Dental infections during pregnancy require special attention in terms of possible complications and treatments due to the potential to affect the lives of two individuals. A case of a 36-year-old pregnant patient with a submandibular abscess caused by an odontogenic infection is reported, followed by a comprehensive systematic review of the literature in order to retrieve information regarding severe odontogenic infections and adverse pregnancy outcomes. The review was conducted according to the PRISMA guidelines using PubMed, Scopus, and Google Scholar databases. A total of 69 cases were included in the qualitative analysis. The mean age was 27.72 years. Patients were managed with surgery in combination with antibiotics. Nine infectious-related cesarean sections were detected, and preterm birth was associated in 3 cases, low birth weight in 2 cases, death of the fetus in 9 cases (13%), and maternal death in 4 cases (5.8%). The possible compromise of oral health during pregnancy is well known; however, severe odontogenic infections are rarely considered in the literature, and they may be associated with severe and life-threatening complications for both mother and the fetus.

## 1. Introduction

Odontogenic infections can potentially develop into deep neck space infections and have severe consequences, with possible life-threatening complications. Common periodontal diseases or dental decays, if not treated or badly treated, can lead to critical infectious disease processes and cause severe consequences such as periodontal or periapical abscess, facial cellulitis, deep neck infections (DNI), Ludwig’s angina, mediastinitis, and cerebral abscess [1]. Oral health is compromised during pregnancy due to all the hormonal and non-hormonal changes in the oral environment [2]. Odontogenic infections and their complications have potentially harmful effects on pregnant women and their developing fetuses and have been associated with several poor pregnancy outcomes [2]. Wong et al., in their paper, reported a total of 346 cases of women with odontogenic infections complicated in ten years, including 5 (1.44%) during pregnancy [2]. In a paper by Omeje et al., in five years, 131 patients were admitted with the diagnosis of cervicofacial cellulitis, of which 21 were pregnant [3]. The possible compromise of oral health during pregnancy is well known; however, severe odontogenic infections are rarely considered in the literature. Pregnancy causes many changes in the physiology of the female patient. Physiologic changes during pregnancy may result in significant variations in the oral microbial flora and may promote the colonization of various pathogens [4]. The main role of hormones in the development of pathologic conditions in the oral cavity is substantial [5]. The rise in circulating levels of estrogen and progesterone can cause the onset of many modifications, such as the alterations of the gingival vascular system, the immune response, the chemical composition, the pH levels, and the normal sub-gingival floor [6]. Periodontal pathogenic species, as well as cariogenic species in this period, can lead to particular conditions such as pregnancy gingivitis, periodontitis, tooth erosion, and dental caries [6]. Therapy for severe neck infections is always challenging, and it’s even more crucial during pregnancy because of the possible life-threatening condition for both the mother and the fetus [7]. The management and the treatment of complicated odontogenic infections are mandatory and can have serious consequences if not done promptly, such as airways compromission, which may also require emergency tracheostomy [6]. Further complications can show up during pregnancy, such as preterm delivery, low birth weight of the baby, as well as the death of the mother and/or the fetus [8]. Doumbia-Singare et al. and Osunde et al. reported an incidence of fetal death of 40% and 30%, respectively, and a maternal death rate of 25% in both studies [8,9]. The purpose of this systematic review is to evaluate the dental origin and treatment of odontogenic severe infections in pregnant patients and the type and frequency of adverse complications. Additionally, it’s important to emphasize that there is not a systematic review that describes and quantifies serious complications for both the fetus and the mother already present in the literature.

## 2. Materials and Methods

### 2.1. Clinical Case

**Figure 1:**
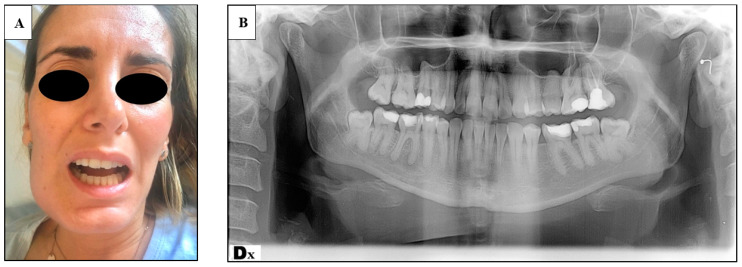
(**A**) Frontal view of the patient presenting submandibular swelling and trismus; (**B**) orthopantomography of the patient showing periapical abscess of the 4.6 tooth.

A 36-year-old and 30 weeks pregnant (multipara) woman referred to the ER of the Policlinico Umberto I, presenting: trismus, submandibular right tumefaction, facial swelling and pain, no dysphagia, no dyspnea (Figure 1A). Her body temperature was 37.8. The oral examination showed a deep carious lesion of the 4.6 (lower right first molar) tooth (Figure 1B). The patient had already been given antibiotic treatment with amoxicillin 1 g two times per day for 5 days by her general dentist. A new antibiotic treatment was set with intravenous parenteral piperacillin, and tazobactam therapy and the patient was admitted to the Department of Obstetrics and Gynecology. After 3 days the patient was taken into a surgical intervention involving the extraction of the tooth and the extraoral drainage of the abscess under local anesthesia. A Penrose drain was left in place for four days. Maternal and fetal parameters were monitored during hospitalization and the surgical procedure. No signs of fetal distress were ever detected. Besides good locoregional conditions, nine days after surgical intervention, the patient went through preterm placental abruption for which a cesarean delivery at 31.6 weeks of pregnancy was necessary. The weight of the baby at birth was 1642 kg, and she was sent to neonatal intensive unit care, where she was intubated and treated for respiratory distress due to lung immaturity. The total hospitalization time of the baby was 12 days. The patient was treated promptly by setting up an adequate antibiotic therapy and performing the extraction and surgical drainage of the abscess, but the patient reported preterm placental abruption anyway, followed by cesarean delivery at 31.6 weeks, and the need of the newborn of an intensive care unit admission.

### 2.2. Systematic Literature Review

A systematic review was conducted in agreement with the PRISMA guidelines [10] in August 2020. The review relates to pregnant women (P), treatment provided (I); pregnant women without odontogenic infections (C) and adverse pregnancy outcomes (O) were evaluated. A systematic comprehensive search of electronic media was performed independently by two authors (R.P., D.D.C.), and any discordance was solved by consensus with a third author (A.C.). Databases used were PubMed, Ovid, Scopus, and ScienceDirect; search keywords included: Ludwig’s angina OR head and neck abscess OR submandibular abscess OR cervicofacial cellulitis OR deep neck infection OR odontogenic infection OR submandibular cellulitis OR maxillofacial infection in combination with pregnancy OR foetal distress OR fetal distress OR fetal deaths OR pregnant OR Intrauterine Fetal Demise OR preterm birth OR premature rupture of membranes. The references identified within the papers were analyzed, and a manual search was conducted for further studies not located in the above-mentioned searches in order to identify any missing publications. Due to the rarity in the literature of these types of complications, case reports and articles not written in English were also included in the review. The study design was not considered as an exclusion criterion given the lack of publications concerning this subject. Works whose full text was not available were excluded. All articles were reviewed for mean age, gestation week, diagnosis on admission, surgical treatment performed, type of anesthesia, antibiotic treatment set, and adverse outcomes. Adverse outcomes recorded were related to the mother or the fetus or both: mother and/or fetal death, sepsis, fetal distress, emergency cesarean section, preterm birth, and low birth weight. The papers included after the final evaluation round were then appraised for quality according to the JBI Critical Appraisal Checklist to assess their risk of bias [11], showed in Table 1 and Table 2.

## 3. Results

**Figure 2:**
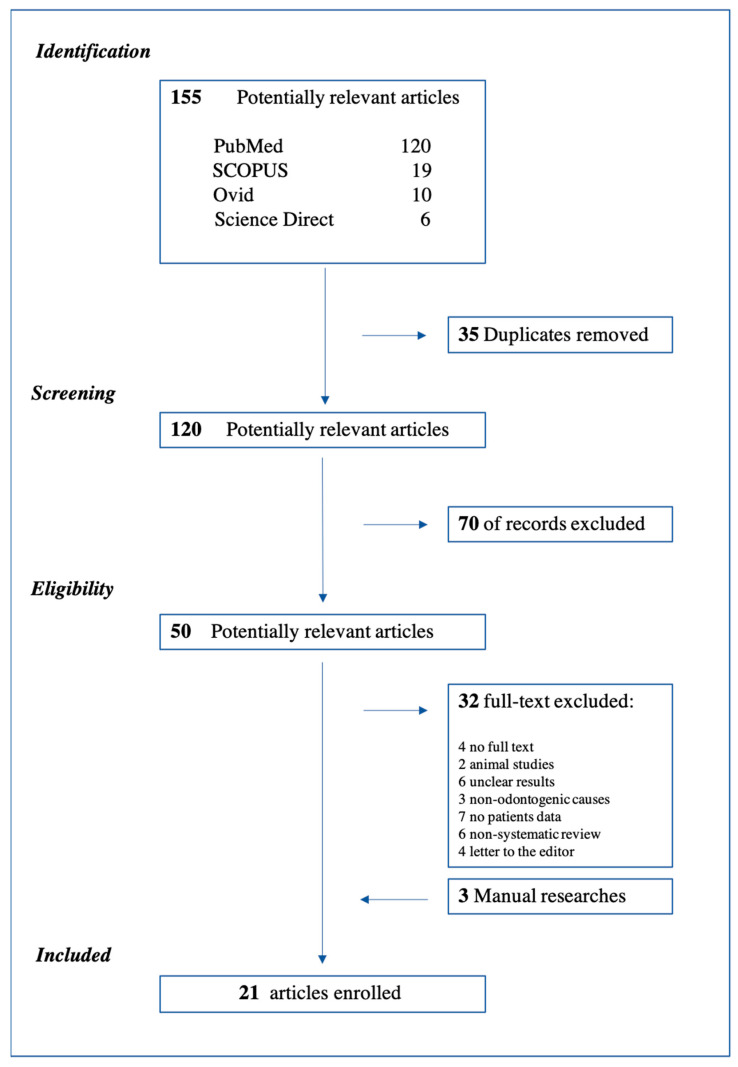
Flow Chart of the study selection. Systematic review was conducted in adherence to PRISMA guidelines.

A total of 155 articles were identified during the first search and 120 after the removal of duplicates. After title and abstract screening, 70 of these 120 articles were excluded as not relevant. The full text of the remaining 50 papers was read and evaluated for eligibility, and 21 papers were included in the study for a total of 69 patients. The flow chart with the details of the screening and selection process of the articles is shown in Figure 2, in accordance with the PRISMA guidelines. Before the case presented above, a total of 69 cases had been described in the literature since 1994. A total of 16 case reports [7,12,13,14,15,16,17,18,19,20,21,22,23,24,25,26] and 5 retrospective studies [2,3,8,9,27] were included in this analysis (Table 1 and Table 2). The mean age of the sample was 27.72 years. In total, 69.5% of the patients came from African countries (Nigeria, Sudan, Mali), 7.2% from India and Hong Kong, respectively, 5.7% from the USA. One case report from France, 1 from Austria, and 1 from Turkey were included in the systematic review. Patients were in the third trimester of pregnancy in 68.1% of cases for a total of 47 cases, in the second trimester in 24.6% (17 patients), and 5 patients developed severe odontogenic complications in the first trimester (7.3%). The most frequent odontogenic cause, when specified, was the carious process of the third molar (upper or lower) in 23% of cases, followed by caries of the first molar in 8.7%, and then second molars and premolars, both with 7.3%. Severe odontogenic complications occurred in this study were: cervicofacial cellulitis in 30% of cases [3,22], Ludwig’s angina in 27.5% [9,14,15,16,18,19,26,27], abscesses (submandibular and submental) in 23.2% [2,12,13,21,27], deep neck infection (DNI) in 21.7% [7,8,17,23,24,25], mediastinitis in 2.3% [7,23], and brain abscess in one case [20]. Sixty-nine patients were included; in one case drainage occurred spontaneously, and in two patients, medical therapy was sufficient to solve the clinical condition. Sixty-six surgical procedures were performed, of which 52 in local anesthesia, 78.8% (one block and one under intravenous sedation), and 14 in general anesthesia, 21.2%. Tracheostomy was necessary in three cases (4.3%) [14,17,19]. Extraoral surgical drainage with the extraction of the dental element was performed in 63.7% of the cases; in 12 cases the extraction was delayed but performed anyway before discharge. A patient with a brain abscess underwent a left-sided hemicraniectomy with the evacuation of subdural empyema, and then, due to a worsening of clinical conditions, she was operated five more times [20]. At last, the patient reported neurological deficits, which included Broca’s aphasia and apraxia associated with the right hemiplegia [21]. A total of 37.7% of the patients included in the systematic review presented adverse outcomes, which could involve either the fetus or the mother or both. Adverse outcomes recorded were: 9 fetal deaths (13%) [7,8,9,22], 4 babies presented fetal distress (5.8%) [12,13,21,23], and 2 needed intensive care unit (ICU) (2.9%) [19,21]. Four mothers died (5.8%) [8,9], 6 needed postoperative ICU (8.7%) [2,14,15,19,24], and 3 cases developed sepsis (4.3%) [7,8]. Nine infectious-related cesarean sections were detected, and 3 patients showed necrotizing fasciitis of the submandibular region and neck [22,27]. Preterm birth was registered in 3 patients (4.3%) [8,19,23] and low birth weight was registered in two cases (2.9%) [18,19]. All details of the surgical treatment and adverse complications are reported in Table 3, Table 4 and Table 5. Following the criteria of the JBI checklist for case reports and case series, it was possible to provide a quality rating of the articles. The total number of case reports, as shown in Table 1 and Table 2, was 12, results were: 10 case reports were ranked good (score: 6–8), 6 were ranked fair (score: 3–5), and no articles were ranked as poor (score: 0–2). One case series was ranked good (score: 8–10), 4 were ranked fair (score: 4–7), and no articles were ranked as poor (score: 0–3).

## 4. Discussion

A comprehensive systematic review of the pertinent literature was performed in order to retrieve information regarding clinical features and the incidence of adverse pregnancy outcomes. The most frequent odontogenic causes were carious processes affecting the third molar (upper or lower in 23% of the cases), the first molar (8.7%), and the second molars and premolars (7.3%). Pregnancy is a time of relative maternal immunocompromise, making the body more susceptible to infections. Human gingiva has been demonstrated to be a target tissue for estrogen and progesterone increases and estradiol-affected periodontal microvascularization that contribute to alterations of oral tissues [28]. There is also a significant alteration in the bacterial mass of the oral cavity, with a shift toward a more anaerobic flora [4]. In the early and middle stages of pregnancy, the prevalence of P. gingivalis, A. actinomycetemcomitans, P. Intermedia, T. Denticulate significantly increased in comparison with nonpregnant women, with an increased proportion of P. intermedia during pregnancy [29]. All these factors are associated with modification of the diet, frequent carbohydrate-rich meals, and increased dental plaque formation, acid production, decay, and periodontal diseases [30]. These collective changes may pose various challenges in providing dental care for pregnant patients [30]. The most frequent severe complications of dental infections are cervicofacial cellulitis (30%), followed by Ludwig’s angina (27.5%), and submandibular/submental abscesses (23.2%). The management of severe infections with neck and airways involvement was well described by Freeman et al., who recommended a radiological evaluation to identify an eventual progression of the infection in order to reduce the mortality [31]. Due to the risk of evolution towards serious consequences, mother and fetus must be subjected to careful clinical and radiological monitoring. All infections involving the neck and oral floor, such as Ludwig’s angina, can rapidly progress and cause airway obstruction [22]. Often, due to edema and trismus associated with the infection, patients necessitated a fiber optic intubation or, in the most extreme conditions, of a tracheostomy, which was needed in three patients (4.3%). Early recognition of the salient clinical features, right diagnosis, and treatment are essential for an effective resolution of these infections and their complications to decrease morbidity and mortality of both the fetus and the mother. The most used surgical treatment (63.7%) was surgical extraoral drainage with tooth extraction performed simultaneously in 71% of the cases, or delayed in 12 cases, but in every case before the discharge of the patient [6,16,18,22]. These data confirm how the resolution of the odontogenic infectious process is fundamental for the patient’s recovery. A pregnant patient with a dental infection requires special attention due to major modifications in physiology and metabolism and all their implications on the cardiovascular, respiratory, and gastrointestinal systems, as well as the above-mentioned changes in the oral cavity [32]. The surgical procedures performed were 66, of which 52 (78.8%) in local anesthesia (1 cervical plexus block and one with intravenous sedation) and 14 (21.2%) in general anesthesia. Three patients required a tracheostomy (4.3%) [14,17,19]. The 37.7% of the patients included in the systematic review presented adverse outcomes involving either the fetus or the mother. Adverse outcomes recorded were: 9 fetal deaths (13%), and 4 patients died (5.8%). Six mothers (8.7%) and 2 babies (2.9%) needed postoperative ICU, and 4 babies presented fetal distress (5.8%). Preterm birth was registered in 3 patients (4.3%); low birth weight was registered in two cases (2.9%). Dental infections, as reported in the literature, can cause disastrous complications, even fetal death [7,8,9]. The incidence of fetal and maternal death registered is high, 13% and 5.8%, respectively. This data must capture attention and raise awareness of the importance of multidisciplinary treatment of these kinds of patients. Frequently a delay in starting a treatment or an ineffective therapeutic approach have been detected, causing progress of the infectious process [15,16,21]. The treatment of the pregnant patient has the potential to affect the lives of two individuals (the mother and the fetus). Certainly, this dogma must never be forgotten so that the treatments performed bring the greatest benefit to the mother while minimizing the risks for the fetus [33]. Dentists could be afraid of performing dental treatments or administering medication during pregnancy because of the potential interactions with the fetus. Probably the best way in these cases is to start a timely and correct antibiotic therapy and quickly access dental care in order to prevent severe complications. There are many safe antibiotics to use during pregnancy and many treatments that can be made from the second trimester of pregnancy onwards [34]. Ampicillin, amoxicillin, and erythromycin, as well as the first-generation cephalosporins, are the antibiotics of the first choice in case of infectious diseases, and they must be administered following the guidelines to avoid possible side effects [35]. Strafford et al. [36] demonstrated how prenatal screening and oral hygiene are important to avoid adverse complications during pregnancy, but despite the safety of providing pregnant patients with dental care, a lack of confidence of dentists, physicians, and patients still exists. The study demonstrated also that the main limitation was financial: only 44% of the patients had received dental treatment [36]. The knowledge of the potential adverse outcomes of odontogenic infections points out a lack of screening during pregnancy. Improving oral health before pregnancy and setting promptly the right treatment could prevent potential complications. Of course, further preventive measures before and during pregnancy are needed. Dentists, head and neck surgeons, gynecologists, and anesthesiologists should be aware of the importance of dental health during pregnancy.

## 5. Conclusions

Even if only case reports and retrospective studies have been included in the study, what is shown is that the complications rate in these kinds of patients is very high. Despite differences in the distribution in the various countries, the mortality rate is substantial.

## Figures and Tables

**Table 1:** Characteristics of included case reports.

	Author, Year, Country of Origin	Title	Journal	Age	Gestation Week	Diagnosis	Risk of Bias *
1	Fayek SS et al. [11] 1994, Saudi Arabia	Awake fibre optic intubation in a 38-week pregnant patient with submandibular abscess.	*Int J. Obstet Anesth.*	32	38 weeks	submandibular abscess	G
2	Martin F. et al. [12] 2004, France	Do we have to monitor foetal heart rate during general anesthesia?	*Ann Fr Anesth Rian*	29	36 weeks	submandibular abscess	F
3	Abramowicz et al. [13] 2006, USA	Severe life-threatening maxillofacial infection in pregnancy presented as Ludwig’s angina.	*Infect Dis Obstet Gynecol.*	24	29 weeks	Ludwig’s angina	G
4	Niederhauser A. et al. [14] 2006, USA	Ludwig’s angina in pregnancy.	*J. Matern Fetal Neonatal Med.*	24	twin gestation at 22 weeks	bilateral Ludwig’s angina	G
5	Soltero R. et al. [15] 2009, Puerto Rico	Successful conservative management of Ludwig’s angina in advanced pregnancy.	*Bol Asoc Med P R.*	20	32 weeks	Ludwig’s angina	F
6	Rana AS. et al. [16] 2009, India	A rare case of simultaneous surgery of an odontogenic space infection and delivery by caesarean section in a pregnant patient.	*J. Maxillofac Oral Surg.*	34	full term pregnant	DNI	G
7	Rajeev S. et al. [17] 2008, India	Anaesthetic management of Ludwig’s angina in pregnancy.	*Int J. Obstet Anesth*	26	32 weeks	Ludwig’s angina	G
8	Moorhead K. et al. [18] 2010, USA	Pregnancy Complicated by Ludwig’s Angina Requiring Delivery.	*Infect Dis Obstet Gynecol.*	24	33 weeks	Ludwig’s Angina + septis and ARDS	G
9	Hobson DT. et al. [19] 2011, USA	Pregnancy complicated by recurrent brain abscess after extraction of an infected tooth.	*Obstet Gynecol.*	35	22 weeks	acute meningoencephalitis and left pterygoids muscles abscess and brain abscess	G
10	Çelebi N. et al. [20] 2013, Turkey	Acute fetal distress following tooth extraction and abscess drainage in a pregnant patient with maxillofacial infection.	*Aust Dent J.*	28	36 weeks	submandibular abscess	G
11	Mukherjee S. et al. [21] 2013, India	Poor Dental Hygiene in Pregnancy Leading to Submandibular Cellulitis and Intrauterine Fetal Demise: Case Report and Literature Review.	*Int J. Prev Med*	38	34 weeks	bilateral submandibular cellulitis and necrosis	F
12	Dalla Torre D. et al. [7] 2014, Austria	Odontogenic deep neck space infection as life-threatening condition in pregnancy.	*Aust Dent J.*	25	28 weeks	DNI mediastinitis and sepsis	F
13	Kamath AT. et al. [22] 2015, India	Ludwig’s Angina in Pregnancy Necessitating Pre Mature Delivery.	*J. Maxillofac Oral Surg.*	24	32 weeks	Ludwig’s angina DNI mediastinitis and sepsis	F
14	Tocaciu S. et al. [23] 2017, Australia	Severe odontogenic infection in pregnancy: a timely reminder.	*Aust Dent J.*	29	17 weeks	odontogenic infection + DNI	F
15	Pereira RDS. et al. [24] 2017, Brazil	Dental Infection and Pregnancy: The Lack of Treatment by the Dental Professional Evolving to a Complex Maxillofacial Infection.	*J. Craniofac Surg.*	30	32 weeks	odontogenic infection + DNI	G
16	Rahman T. et al. [25] 2019, India	Decompression of Ludwig’s angina in a pregnant patient under bilateral superficial cervical plexus block.	*J. Perioper Pract*	25	28 weeks	Ludwing’s Angina	G

DNI: deep neck infection. * JBI Critical Appraisal Checklist to assess their risk of bias. Quality Rating: poor 0–2; fair 3–5; good 6–8 [11].

**Table 2:** Characteristics of included retrospective studies.

	Author, Year, Country of Origin	Title	Journal	Patients Included	Age	Gestation Week	Diagnosis	Risk of Bias *
17	Wong D et al. [2] 2012, Hong Kong	Management of severe odontogenic infections in pregnancy.	*Aust Dent J.*	5	33; 32; 22; 26; 29	33; 35; 8; 10; 30 weeks	dental abscess	F
18	Doumbia-Singare K. et al. [8] 2014, Mali	Cervico-facial cellulitis during pregnancy: about a series of 10 cases in Mali.	*Bull Soc Pathol Exot.*	10	23 (range 16–31)	1 first trimester; 6 second and 3 third trimester	odontogenic infection + DNI	F
19	Osunde O et al. [9] 2014, Nigeria	Management of Ludwig’s Angina in Pregnancy: A Review of 10 Cases.	*Ann Med Health Sci Res.*	10	29.5 years SD ± 5.3	third trimester	Ludwing’s angina	G
20	Ali EAM. et al. [26] 2019, Sudan	Delay in the Referral of Pregnant Patients with Fascial Spaces Infection: A Cross-Sectional Observational Study from Khartoum Teaching Dental Hospital, Sudan.	*J. Maxillofac. Oral Surg.*	10	26.5 years SD ± 6.3	6 third trimester; 4 second trimester	7 submandibular abscess; 1 submental abscess; 2 Ludwig’s angina	F
21	Omeje KU. et al. [3] 2020, Nigeria	Severe Cervicofacial Cellulitis in Pregnancy—A Review of 18 Cases.	*Iran J. Otorhinolaryngol.*	18	29 years SD ± 7.1	12 third trimester; 4 second; 2 in the first trimester	Cervicofacial cellulitis	F

DNI: deep neck infection. * JBI Critical Appraisal Checklist to assess their risk of bias. Quality Rating: poor 0–3; fair 4–7; good 8–10 [11].

**Table 3:** Summary of odontogenic cause, surgical treatment, and adverse outcomes.

	Author, Year, Country of Origin	Odontogenic Cause	Intervention	Anaesthesia	Adverse Outcomes
1	Fayek SS et al. [11] 1994, Saudi Arabia	right second molar tooth in the lower jaw	drainage + extraction	GA	fetal distress and C-section 38 weeks
2	Martin F. et al. [12] 2004, France	dental abscess NR	drainage	GA	foetal distress and C-section 36 weeks
3	Abramowicz et al. [13] 2006, USA	lower left third molar	awake tracheostomy + drainage + extraction	GA	maternal ICU post op (6 days)
4	Niederhauser A. et al. [14] 2006, USA	multiple periapical abscesses	bilateral drainage + multiple extractions	GA	maternal ICU post op (1 day)
5	Soltero R. et al. [15] 2009, Puerto Rico	recurrence of a periapical abscess	drainage + extraction	LA	no complications
6	Rana AS. et al. [16] 2009, India	lower right third molar	tracheostomy +drainage + delay extraction	GA	C-section 34 weeks simultaneously with the surgical procedure
7	Rajeev S. et al. [17] 2008, India	right second molar	drainage	GA	normal delivery 37 weeks of a 1700-g baby
8	Moorhead K. et al. [18] 2010, USA	tooth abscess NR	tracheotomy + drainage + extraction of five of teeth	GA	preterm C-section; infant weighing 2120 grams: mather and baby needed ICU
9	Hobson DT. et al. [19] 2011, USA	left maxillary third molar	drainage of left pterygoid abscess + multiple partial lobectomies	GA	C-section at 39 weeks; maternal neurologic deficits, which included Broca’s aphasia and apraxia with right hemiplegia
10	Çelebi N. et al. [20] 2013, Turkey	lower left third molar	drainage + extraction	LA	8 h later foetal distress: C-section, baby needed ICU (12 days)
11	Mukherjee S. et al. [21] 2013, India	second or third mandibular molar	drainage	GA	fetal death normal delivery 34 weeks and maternal necrotizing fasciitis
12	Dalla Torre D. et al. [7] 2014, Austria	first and second lower left molars	drainage + extraction	GA	mathernal sepsis and ARDS, intrauterine death of the foetus and C-section at 28 weeks
13	Kamath AT. et al. [22] 2015, India	lower first molar and right upper second molar	bilateral drainage + dentals extraction and second neck and mediastinum drainage	GA	foetal distress C-section 32 weeks preterm delivery
14	Tocaciu S. et al. [23] 2017, Australia	lower left third molar	drainage + extraction	GA	maternal ICU post op (3 days)
15	Pereira RDS. et al. [24] 2017, Brazil	lower left third molar	drainage + delay extraction	LA	no complications
16	Rahman T. et al. [25] 2019, India	lower left second molar	drainage + extraction.	LA + superficial cervical plexus block	no complications

NR: not reported; GA: general anesthesia; LA: local anesthesia; C-section: cesarean section; ICU: intensive care unit.

**Table 4:** Summary of odontogenic cause, surgical treatment, and adverse outcomes.

	Author, Year, Country of Origin	Odontogenic Cause	Intervention	Anaesthesia	Adverse Outcomes
17	Wong D et al. [2] 2012, Hong Kong	5 dental abscess	4 drainage + 2 contemporary extractions, 1 spontaneus drainage	2 GA; 1 LA; LA+IV sedation	2 patients required post op ICU, 1 C-section (patient has previous C-sections); 1 planned abortion.
18	Doumbia-Singare K. et al. [8] 2014, Mali	5 third molar tooth; 5 premolar tooth	8 drainage 2 medical treatment only	LA	1 premature birth, 4 fetus deaths, 2 maternal deaths caused by sepsis
19	Osunde O et al. [9] 2014, Nigeria	8 odontogenic infection; 2 unknown	drainage	LA	3 fetal deaths, 2 maternal deaths, of which only one death occurred in hospital.
20	Ali EAM. et al. [26] 2019, Sudan	5 lower third molar; 4 lower first molars; 1 lower canine	drainage and extractions	LA	2 patients with necrotizing fasciitis
21	Omeje KU. et al. [3] 2020, Nigeria	odontogenic infection	drainage + 8 extractions, 10 delay extractions	LA	NR

NR: not reported; GA: general anesthesia; LA: local anesthesia; C-section: cesarean section; ICU: intensive care unit.

**Table 5:** Sample characteristics. Summary of the results.

	Sample Characteristics	N.
**Age (Mean +/** **− SD)**	27.72 years +/− 5.3	69
**Gestational age**	First trimester	7.3%
	Second trimester	24.6%
	Third trimester	68.1%
**Nationality**	African countries (Nigeria, Sudan, Mali)	69.5%
	India	7.2%
	Hong Kong	7.2%
	USA	5.7%
**Odontogenic cause**	Third molar	23%
	First molar	8.7%
	Second molars	7.3%
	Premolars	7.3%
**Surgical treatment**		
	Extraoral surgical drainage with extraction	63%
	Extraoral surgical drainage + delay extraction	17%
	General anesthesia	21.2%
	Local anesthesia	78.8%
**Odontogenic complications**		
	Cervicofacial cellulitis	30%
	Ludwig’s angina	27.5%
	Abscesses (submandibular and submental)	23.2%
	Deep neck infection (DNI)	21.7%
	Mediastinitis	2.3%
**Adverse outcomes**		
	Fetal deaths	13%
	Fetal distress	5.8%
	Baby needs of ICU	2.9%
	Preterm birth	4.3%
	Low birth weight	2.9%
	Maternal deaths	5.8%
	Mother need of ICU	8.7%
	Infection-related C-section	13%

ICU: Intensive Care Unit.

## Data Availability

The data presented in this study are available on request from the corresponding author.

## References

[1] Weise H., Naros A., Weise C., Reinert S., Hoefert S. (2019). Severe odontogenic infections with septic progress—A constant and increasing challenge: A retrospective analysis. BMC Oral Health.

[2] Wong D., Cheng A., Kunchur R., Lam S., Sambrook P., Goss A. (2012). Management of severe odontogenic infections in pregnancy. Aust. Dent. J..

[3] Omeje U., Omeje I., Agbara R. (2020). Severe Cervicofacial Cellulitis in Pregnancy—A Review of 18 Cases. Iran. J. Otorhinolaryngol..

[4] Nuriel-Ohayon M., Neuman H., Koren O. (2016). Microbial Changes during Pregnancy, Birth, and Infancy. Front. Microbiol..

[5] Amar S., Chung K.M. (1994). Influence of hormonal variation on the periodontium in women. Periodontology 2000.

[6] Villa A., Abati S., Pileri P., Calabrese S., Capobianco G., Strohmenger L., Ottolenghi L., Cetin I., Campus G.G. (2013). Oral health and oral diseases in pregnancy: A multicentre survey of Italian postpartum women. Aust. Dent. J..

[7] Dalla Torre D., Burtscher D., Höfer D., Kloss F.R. (2014). Odontogenic deep neck space infection as life-threatening condition in pregnancy. Aust. Dent. J..

[8] Doumbia-Singare K., Timbo S.K., Keita M., Mohamed A.A., Guindo B., Soumaoro S. (2014). Cellulite cervico-faciale au cours de la grossesse. À propos d’une série de 10 cas au Mali [Cervico-facial cellulitis during pregnancy: About a series of 10 cases in Mali]. Bull. Soc. Pathol. Exot..

[9] Osunde O., Bassey G., Ver-Or N. (2014). Management of Ludwig’s Angina in Pregnancy: A Review of 10 Cases. Ann. Med. Health Sci. Res..

[10] Moher D., Liberati A., Tetzlaff J., Altman D.G., PRISMA Group (2009). Preferred reporting items for systematic reviews and meta-analyses: The PRISMA statement. PLoS Med..

[11] Moola S., Munn Z., Tufanaru C., Aromataris E., Sears K., Sfetc R., Currie M., Lisy K., Qureshi R., Mattis P., Aromataris E., Munn Z. (2020). Chapter 7: Systematic reviews of etiology and risk. JBI Manual for Evidence Synthesis.

[12] Fayek S., Isaac P., Shah J. (1994). Awake fibre optic intubation in a 38-week pregnant patient with submandibular abscess. Int. J. Obstet. Anesth..

[13] Martin F., Viviand X., Desbriere R., Boubli L., Martin C. (2004). Faut-il monitorer le rythme cardiaque foetal au cours d’une anesthésie générale? [Do we have to monitor foetal heart rate during general anesthesia?]. Ann. Françaises d’Anesthésie Réanimation.

[14] Abramowicz S., Abramowicz J.S., Dolwick M.F. (2006). Severe life threatening maxillofacial infection in pregnancy presented as Ludwig’s angina. Infect. Dis. Obstet. Gynecol..

[15] Niederhauser A., Kirkwood D., Magann E.F., Mullin P.M., Morrison J.C. (2006). Ludwig’s angina in pregnancy. J. Matern. Neonatal Med..

[16] Soltero R., Mercado-Alvarado J. (2009). Successful conservative management of Ludwig’s angina in advanced pregnancy. Bol. Asoc. Med. Puerto Rico.

[17] Rana A.S., Lall S., Kala G., Tyagi A. (2009). A rare case of simultaneous surgery of an odontogenic space infection and delivery by caesarean section in a pregnant patient. J. Maxillofac. Oral Surg..

[18] Rajeev S., Panda N.B., Batra Y.K. (2009). Anaesthetic management of Ludwig’s angina in pregnancy. Int. J. Obstet. Anesth..

[19] Moorhead K., Guiahi M. (2010). Pregnancy complicated by Ludwig’s angina requiring delivery. Infect. Dis. Obstet. Gynecol..

[20] Hobson D.T.G., Imudia A.N., Soto E., Awonuga A.O. (2011). Pregnancy complicated by recurrent brain abscess after extraction of an infected tooth. Obstet. Gynecol..

[21] Çelebi N., Kütük M., Tas M., Soylu E., Etöz O., Alkan A. (2013). Acute fetal distress following tooth extraction and abscess drainage in a pregnant patient with maxillofacial infection. Aust. Dent. J..

[22] Mukherjee S., Sharma S., Maru L. (2013). Poor dental hygiene in pregnancy leading to submandibular cellulitis and intrauterine fetal demise: Case report and literature review. Int. J. Prev. Med..

[23] Kamath A.T., Bhagania M.K., Balakrishna R., Sevagur G.K., Amar R. (2012). Ludwig’s Angina in Pregnancy Necessitating Pre Mature Delivery. J. Maxillofac. Oral Surg..

[24] Tocaciu S., Robinson B.W., Sambrook P.J. (2017). Severe odontogenic infection in pregnancy: A timely reminder. Aust. Dent. J..

[25] Pereira R.D.S., Gomes-Ferreira P.H.S., Bonardi J.P., Da Silva J.R., Latini G.L., Hochuli-Vieira E. (2017). Dental Infection and Pregnancy: The Lack of Treatment by the Dental Professional Evolving to a Complex Maxillofacial Infection. J. Craniofacial Surg..

[26] Rahman T., Ahmed S., Rahman S. (2019). Decompression of Ludwig’s angina in a pregnant patient under bilateral superficial cervical plexus block. J. Perioper. Pract..

[27] Ali E.A.M., Eltayeb A.S., Osman M.A.K. (2020). Delay in the Referral of Pregnant Patients with Fascial Spaces Infection: A Cross-Sectional Observational Study from Khartoum Teaching Dental Hospital, Sudan. J. Maxillofac. Oral Surg..

[28] Romero B.C., Chiquito C.S., Elejalde L.E., Bernardoni C.B. (2002). Relationship between periodontal disease in pregnant women and the nutritional condition of their newborns. J. Periodontol..

[29] Borgo P.V., Rodrigues V.A.A., Feitosa A.C.R., Xavier K.C.B., Avila-Campos M.J. (2014). Association between periodontal condition and subgingival microbiota in women during pregnancy: A longitudinal study. J. Appl. Oral Sci..

[30] Kurien S., Kattimani V.S., Sriram R.R., Sriram S.K., Bhupathi A., Bodduru R.R., Patil N.N. (2013). Management of pregnant patient in dentistry. J. Int. Oral Health.

[31] Freeman R.K., Vallières E., Verrier E.D., Karmy-Jones R., Wood D.E. (2000). Descending necrotizing mediastinitis: An analysis of the effects of serial surgical debridement on patient mortality. J. Thorac. Cardiovasc. Surg..

[32] Giglio J.A., Lanni S.M., Laskin D.M., Giglio N.W. (2009). Oral health care for the pregnant patient. J. Can. Dent. Assoc..

[33] Flynn T.R., Susarla S.M. (2007). Oral and maxillofacial surgery for the pregnant patient. Oral Maxillofac. Surg. Clin. N. Am..

[34] Official Source Ministero della Salute Ministero Della Salute, Raccomandazioni per la Promozione della Salute Orale in Età Perinatale Recommendations for the Promotion of Oral Health in Perinatal Age. http://www.salute.gov.it/imgs/C_17_pubblicazioni_2317_allegato.pdf.

[35] Mylonas I. (2011). Antibiotic chemotherapy during pregnancy and lactation period: Aspects for consideration. Arch. Gynecol. Obstet..

[36] Strafford K.E., Shellhaas C., Hade E. (2008). Provider and patient perceptions about dental care during pregnancy. J. Matern. Neonatal Med..

